# Unraveling the immunogenetic landscape of autism spectrum disorder: a comprehensive bioinformatics approach

**DOI:** 10.3389/fimmu.2024.1347139

**Published:** 2024-04-24

**Authors:** Jieying Ma, Deyang Liu, Jianzhong Zhao, Xiaolu Fang, Dengyin Bu

**Affiliations:** ^1^ Department of Psychiatric Medicine, Xiangyang Central Hospital, Hubei University of Arts and Science, Xiangyang, China; ^2^ Department of Rehabilitation Medicine, Xiangyang No.1 People’s Hospital, Hubei University of Medicine, Xiangyang, China; ^3^ Department of Clinical Laboratory, Xiangyang No.1 People’s Hospital, Hubei University of Medicine, Xiangyang, China

**Keywords:** immune infiltration, characteristic genes, metabolic subclass, autism spectrum disorders, single cells

## Abstract

**Background:**

Autism spectrum disorder (ASD) is a disease characterized by social disorder. Recently, the population affected by ASD has gradually increased around the world. There are great difficulties in diagnosis and treatment at present.

**Methods:**

The ASD datasets were obtained from the Gene Expression Omnibus database and the immune-relevant genes were downloaded from a previously published compilation. Subsequently, we used WGCNA to screen the modules related to the ASD and immune. We also choose the best combination and screen out the core genes from Consensus Machine Learning Driven Signatures (CMLS). Subsequently, we evaluated the genetic correlation between immune cells and ASD used GNOVA. And pleiotropic regions identified by PLACO and CPASSOC between ASD and immune cells. FUMA was used to identify pleiotropic regions, and expression trait loci (EQTL) analysis was used to determine their expression in different tissues and cells. Finally, we use qPCR to detect the gene expression level of the core gene.

**Results:**

We found a close relationship between neutrophils and ASD, and subsequently, CMLS identified a total of 47 potential candidate genes. Secondly, GNOVA showed a significant genetic correlation between neutrophils and ASD, and PLACO and CPASSOC identified a total of 14 pleiotropic regions. We annotated the 14 regions mentioned above and identified a total of 6 potential candidate genes. Through EQTL, we found that the CFLAR gene has a specific expression pattern in neutrophils, suggesting that it may serve as a potential biomarker for ASD and is closely related to its pathogenesis.

**Conclusions:**

In conclusion, our study yields unprecedented insights into the molecular and genetic heterogeneity of ASD through a comprehensive bioinformatics analysis. These valuable findings hold significant implications for tailoring personalized ASD therapies.

## 1 Introduction

Autism Spectrum Disorders (ASD), is a prevalent developmental disorder characterized by impediments in social communication, deficiencies in both verbal and nonverbal communication, restricted interests, and repetitive behaviors (1). In recent times, there has been a gradual rise in the global population affected by ASD. Currently, it is widely acknowledged that the etiology of ASD is multifaceted, encompassing genetic, environmental, and psychological factors, among others (2, 3). Nonetheless, despite extensive research spanning decades, the underlying causes of ASD remain elusive, and the existing treatment modalities are far from adequate, let alone curative.

Although the diagnosis of ASD has posed challenges for an extended period, the existing biomarkers are insufficient to provide personalized gene-level treatment (4, 5). However, molecular subtypes could aid in identifying the heterogeneity among ASD patients and facilitating the discovery of targeted therapies for ASD (6). Presently, numerous studies posit that inflammatory cells play a pivotal role in the pathogenesis of ASD. Extensive clinical and animal research indicates a strong correlation between inflammation and mental illnesses as well as neurodegenerative diseases. Furthermore, certain studies indicate that the activation of inflammatory cells is also related to central nervous system disorders (3, 5, 7). The activation of inflammatory cells is associated with various molecular structures, including cell membrane receptors for central nervous system signaling molecules, cell membrane channels, intracellular signal transduction pathways, and activation of intracellular transcription factors (8). This activation further leads to the release of proinflammatory factors, such as interleukin-1β (IL-1β), interleukin-6 (IL-6), and tumor necrosis factor-α (TNF-α), thereby causing neuronal damage and loss (9, 10). Specifically, the intimate connection between inflammation and ASD can be attributed to the upregulated expression of proinflammatory factors, which closely relates to hippocampal and brain parenchymal damage in newborns.

In this investigation, We first evaluated the immune infiltration of ASD samples. We also utilized the Weighted Correlation Network Analysis R software package to determine the module that was most highly correlated with the ASD and immune cells. To further limit the selection range of feature genes, we employed Consensus Machine Learning Driven Signatures (CMLS). Subsequently, we evaluated the genetic correlation between immune cells and ASD used GNOVA. And pleiotropic genes identified by PLACO and CPASSOC between ASD and immune cells. FUMA was used to identify pleiotropic regions, and expression trait loci (EQTL) analysis was used to determine their expression in different tissues and cells. Finally, we use qPCR to detect the gene expression level of the core gene. In brief, we have successfully identified 6 core genes that exhibit extraordinary diagnostic potential and present themselves as promising therapeutic targets for ASD.

## 2 Materials and methods

### 2.1 Data collection and processing

**Figure 1:**
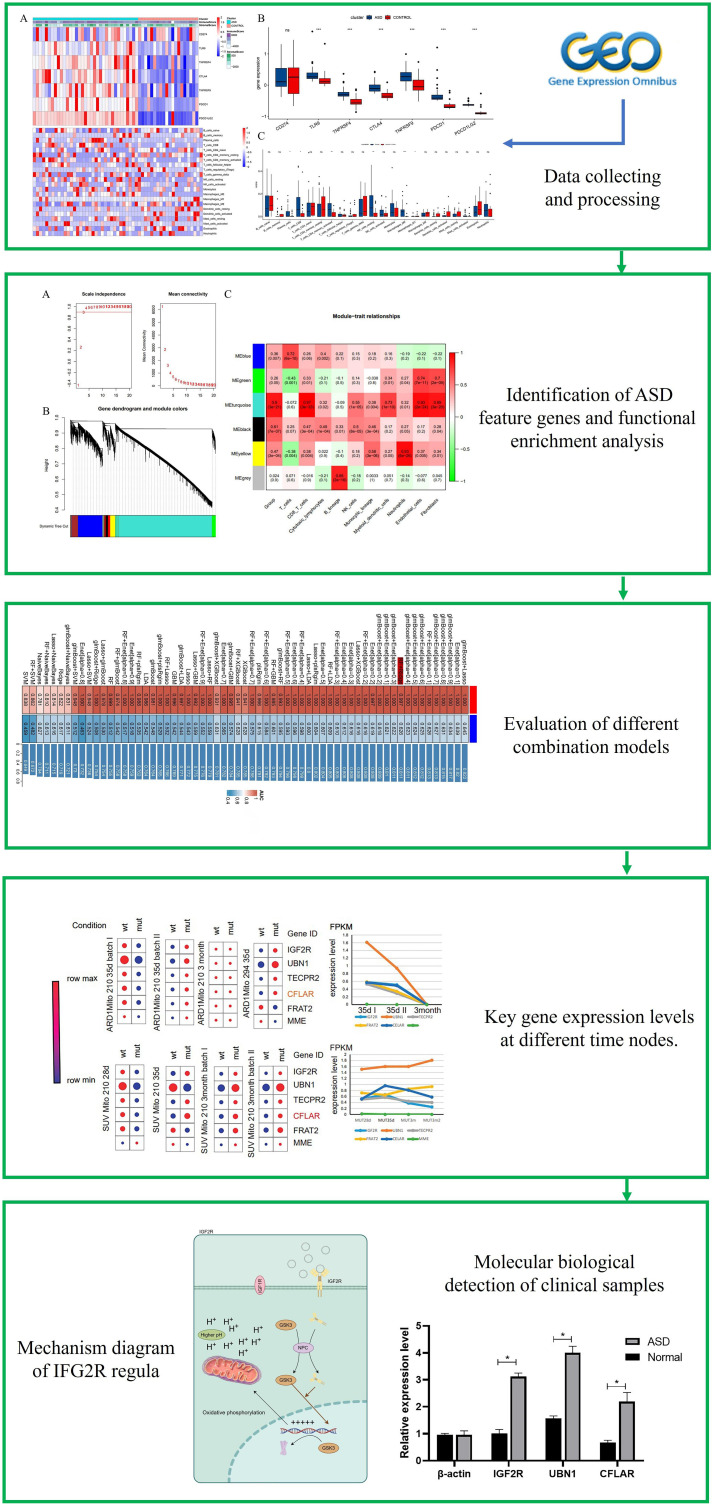
Flowchart of the research.

We devised a comprehensive study flowchart (**Figure 1**). The gene expression matrix of ASD patients involved in this study comes from Gene Expression Omnibus (GEO, https://www.ncbi.nlm.nih.gov/geo/) database created by the National Biotechnology Information Center. After screening, three data sets were included, namely GSE6575, GSE26415 and GSE42133. Subsequently, we first filter the genes recorded in the three data sets and correct the background to ensure that the data will not be disturbed. Subsequently, we logarithmically transformed the data and further normalized it. Finally, the three data sets are properly merged and batch corrected by using the Combat method in the “sva” package. GWAS data acquisition for ASD from the Psychiatric Genomic Consortium (https://www.med.unc.edu/pgc/results-and-downloads).

### 2.2 Evaluation of immune infiltration

In this study, many algorithms were used to calculate the immune infiltration. Firstly, XCELL software package was used to calculate the gene expression profile of ASD and quantify their internal immunity and relative abundance of stromal cells. Subsequent algorithms for evaluating the scores and relative abundance of immune cells come from EPIC (11), ssGSEA (12), quanTIseq (13), TIMER (14), CIBERSORT (15), MCPCounter, XCELL and ESTIMATE.

### 2.3 Weighted correlation network analysis and functional enrichment analysis

We utilized the WGCNA software package to construct the WGCNA network with the aim of identifying gene modules associated with two subclasses of ASD and the clinical features of ASD patients (16). To ascertain the optimal threshold, additional scale-free topological criteria are applied. The cluster tree analysis encompasses over 50 modules, distinguished based on diverse colors. The R software package “Cluster Profiler” is utilized to conduct gene ontology (GO) and Kyoto Gene and Genome Encyclopedia (KEGG) analyses, elucidating the positions and pathways of pivotal genes within the cyan module.

### 2.4 Consensus Machine Learning Driven Signatures

In this study, we used the following R packages (openxlsx, seqinr, plyr, randomForestSRC, glmnet, plsRglm, gbm, caret, mboost, e1071, BART, MASS, snowfall, xgboost, ComplexHeatmap, RColorBrewer, pROC) and harnessed the capabilities of 12 machine learning algorithms. These machines learning included Lasso, Ridge, Enet, Stepglm, SVM, glmBoost, LDA, plsRglm, RandomForest, GBM, XGBoost, NaiveBayes. Importantly, several algorithms, such as RF and Lasso, inherently offer feature selection capabilities. These feature selection capabilities are instrumental in refining our model, allowing it to focus on the most predictive attributes. Therefore, we mainly choose feature variables based on RF or LASSO (17).

In ML framework, the RF model was implemented via the randomForestSRC package. RF had two parameters ntree and rf_nodesize, where ntree represented the number of trees in the forest and rf_nodesize was the number of randomly selected variables for splitting at each node. In this study, we set ntree to 1000 and rf_nodesize to 5 for RF model construction. The Enet, Lasso, and Ridge were implemented via the glmnet package. We utilized the built-in cv.glmnet function from the glmnet package to perform 10-fold cross-validation. This approach was employed to accurately determine the optimal value of the lambda parameter, which is critical for regularization in the Elastic Net model. Additionally, the alpha parameter was dynamically set within the framework to distinguish between the Lasso (alpha = 1) and Ridge (alpha = 0) penalties. The Stepglm was implemented via stats package. A stepwise algorithm using the AIC (Akaike information criterion) was applied, and the direction mode of stepwise search was set to “both”, “backward”, and “forward”, respectively. The SVM was implemented via e1071 package. This approach allows for the construction of a hyperplane or set of hyperplanes in a high-dimensional space, which can be used for classification, regression, or other tasks. Particularly, the SVM implementation in e1071 is versatile and supports various SVM kernels. The GBM was implemented using the gbm package. The gbm function facilitates the construction of the GBM model, which is particularly adept at handling complex nonlinear relationships within high-dimensional datasets. In this analysis, this function was employed to identify the optimal number of trees by minimizing the cross-validation error through an iterative process. The glmboost model was implemented using the mboost package. It employs cross-validation to determine the optimal number of boosting iterations, enhancing model performance while preventing overfitting. The function supports two modes: returning the final model for predictions (“Model” mode) or extracting significant predictors (“Variable” mode), thus facilitating both predictive accuracy and interpretability of influential variables. LDA is implemented in your function using the caret package, emphasizing the use of training data, labels, and cross-validation to improve model performance. The plsRglm was implemented using the plsRglm package. The plsRglm mainly conducts cross-validation with ten folds to optimize model parameters and validate the model’s predictive performance. This approach aims to enhance model accuracy while addressing high-dimensionality and multicollinearity in the dataset. The XGBoost was implemented using the xgboost package. This function performs 5-fold cross-validation to determine the optimal number of boosting rounds by minimizing the test log loss, ensuring a balanced approach between prediction accuracy and model simplicity.The NaiveBayes was implemented using the e1071 package. It constructs the model by combining feature data with the target variable, ensuring the target is treated as a categorical outcome, thus facilitating both predictive modeling and feature analysis. Finally, in ML framework, we employ a novel approach by combining two distinct machine learning algorithms: one for variable selection and the other for model construction. This methodology leverages the strengths of different algorithms to enhance the predictive performance and interpretability of the resulting models.

### 2.5 Estimate genetic correlation with GNOVA

We utilized GNOVA to evaluate the impact of single nucleotide polymorphisms (SNPs) on the genetic inheritance of ASD and neutrophils. This involved conducting regression analysis on the z-statistics produced from two separate studies on these traits. The studies utilized LD scores precomputed using 1000 Genomes European data (18, 19). Through this methodology, we estimated both the heritability (h2) of ASD attributed to SNPs and the overall genetic correlation (rg) between ASD and neutrophils.

### 2.6 PLACO

First, we defined the set of SNPs as those located within a particular gene using the VEGAS annotation file. Then, the P values of the SNPs within each gene are weighted averaged to obtain a gene-level P value, and the P values are simultaneously transformed into Z-statistics. Finally, the newly determined Z-statistic was subjected to a pleiotropy test using the PLACO method. PLACO is an innovative method for detecting pleiotropy at the level of SNPs using the concept of composite null hypothesis from high-dimensional mediation analysis (20). Previous simulations and variance-component-based mediation analyses under the composite null hypothesis have suggested the potential use of this method to assess validity at the gene level (21, 22). Consequently, we used it to identify polymorphic associations at the gene level. To mitigate the impact of excessively large effects, SNPs with extreme Z2 (>80) values were excluded. PLACO assumes three sub-null scenarios for each SNP studied using the composite null hypothesis of pleiotropy: (i) H00: The SNP is not associated with either disease. (ii) H01: The SNP has an effect only on the first disease. (iii) H02: The gene has an effect only on the second disease. (is H1: the SNP effect on both diseases, which represents a pleiotropic relationship.

### 2.7 Pairwise cross-trait meta-analysis using Cross Phenotype Association

A pairwise cross-trait meta-analysis was performed utilizing Cross Phenotype Association (CPASSOC). CPASSOC integrates effect estimates and standard errors obtained from GWAS summary statistics. This approach aims to examine the hypothesis of association between a single nucleotide polymorphism (SNP) and two traits (23). For this analysis, we utilized the heterogeneous version of cross-phenotype association (SHet). SHet employs a fixed-effect model weighted by sample size, rendering it more potent for detecting heterogeneous effects when they exist among studies (24).

### 2.8 Functional analysis for pleiotropic regions

We performed differential expression analysis and gene set enrichment analysis with FUMA for the pleiotropic genes analyzed by PLACO and CPASSOC employing FUMA as the tool (25). expression trait loci (EQTL) analysis will from GTEx and other database were collected for 53 different tissues, with a final consideration of 22,146 genes. The resulting gene sets with adjusted P ≤ 0.05 were reported as significant findings.

### 2.9 Experimental specimen

In order to further study the expression level of the selected target genes in ASD and normal people, we collected new blood samples (10ml, EDTA anticoagulation) from ASD children and normal children, with 5 people in each group. Subsequently, the collected blood was stored at 4 degrees, and serum was obtained by low-speed centrifugation. The people included in this study are volunteers, who voluntarily provide blood samples after being fully explained, and pass the examination and approval of the ethics Committee of the hospital.

### 2.10 Quantitative reverse−transcription polymerase chain reaction

In this research, we included 5 ASD and 5 healthy individuals from Xiangyang No.1 People’s Hospital for qPCR analysis. The gathered blood was subjected to centrifugation at a speed of 3000 revolutions per minute for a duration of 10 minutes. Subsequently, the upper portion of the resulting serum was preserved for mRNA extraction. The Trizol reagent (Life Technologies, USA) was employed to extract the total RNA from the serum. Afterwards, cDNA synthesis was conducted using the RevertAid First Strand cDNA synthesis kit (Fermentas, Canada) with the isolated total RNA. PCR amplification was executed using the QuantiTect SYBR Green PCR kit (Qiagen, Inc) utilizing the ABI Prism 7000 sequence detection system (Applied Biosystem, CA, USA). The cycling conditions were as follows: an initial denaturation step at 95°C for 10 minutes, followed by 40 cycles of denaturation at 95°C for 15 seconds and annealing/extension at 60°C for 1 minute. The mRNA expression level was determined employing the 2^-△△Ct^ method, and subsequently normalized to the expression level of β-actin. All experiments were repeated five times, and the primer sequences are listed in **Table 1**.

**Table 1:** RT-qPCR primer sequences.

Genes	primer sequences
β-actin	F: CATGTACGTTGCTATCCAGGO
	R: ATCCTTAATGTCACGCACGAT
IGF2R	F: GTGACCAGCAAGGCACAAATO
	R: CACCAAGTAGGCACCACTAAG
UBN1	F: CCTGAATCCTGCGTTTTTGAAG
	R: GCAGCGTTTGTGATCTGGTT
CFLAR	F: TCAAGGAGCAGGGACAAGTTA
	R: GACAATGGGCATAGGGTGTTATC

### 2.11 Statistical analysis

R language (version 4.2.0) is used for statistical analysis. Wilcoxon test was used for comparison between groups. *P*-value < 0.05 were considered as potential associations.

## 3 Results

### 3.1 Association between the ASD and immune infiltration

**Figure 2:**
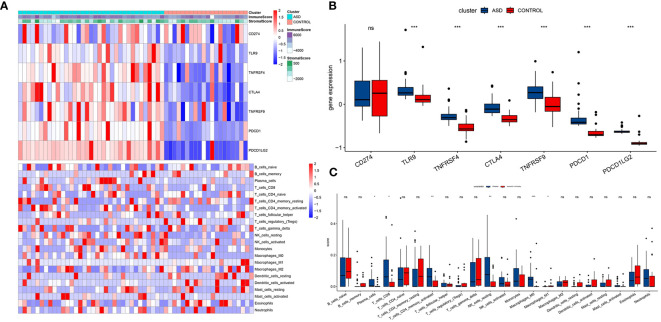
Association between ASD subclasses and immune infiltration. **(A)** Heatmap describing the immune infiltration landscape in the two ASD subclasses. **(B)** Boxplots describing the distribution of expression for the immune. **(C)** TME cells signatures (ns indicates no significance, *P < 0.05, **P < 0.01, ***P < 0.005).

The flowchart systematically described our study (**Figure 1**). The essence of using the ESTIMATE algorithm to determine the subgroup characteristics of ASD is to achieve the goal by scoring immunity and cell matrix. Interestingly, there was a significant difference in the immune score between the two groups, while the ASD group showed a higher matrix score (**Figure 2A**). In order to further clarify immune infiltration and understand the immune characterization, we quantified the abundance of 22 kinds of immune cells in the microenvironment (**Figure 2B**). Several immune checkpoint genes found in the ASD group showed higher levels, suggesting their potential as immunotherapy targets in the future, including TLR9, TNFRSF4, CTLA4, TNFRSF9, PDCD1 and PDCD1LG2 (**Figure 2C**). In addition, ASD showed different expression levels in six immune cell subgroups, including Plasma cells, CD8 cells, activated CD4 cells, resting NK cells, Macrophages M0 and Macrophages M1. It is worth noting that, unlike the significantly increased gene expression, macrophages of immune cell subgroup M1 showed significantly high infiltration, but M2 showed significantly low infiltration risk. Notably, unlike the significantly increased gene expression, macrophages of immune cell subgroup M1 showed significantly high infiltration, but M2 showed significantly low infiltration risk.

### 3.2 WGCNA

**Figure 3:**
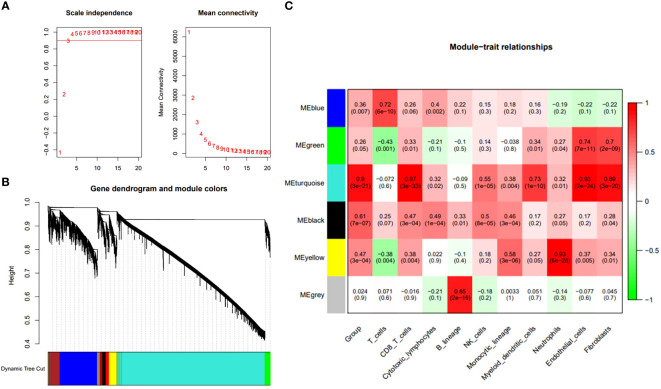
Identification of ASD feature genes and functional enrichment analysis. **(A)** The interconnection between network genes shows scale-free network distribution under soft threshold power. **(B)** Identification of co-expression gene modules. The branches of the dendrogram clustered into six modules, each labeled with a unique color. **(C)** A heat map showing the correlation between modules and characteristic gene sets.

We further applied WGCNA depth analysis to the merged data set to extract the clinical phenotype related to ASD and immune cells. When the threshold is 4, the scale-free network and connectivity show the greatest compatibility (**Figure 3A**). Further clustering tree algorithm divides ASD-related genes into six gene modules, and each module is represented by a special color (**Figure 3B**). Among them, cyan modules contain the most genes, and have the most significant correlation with the poor prognosis of ASD (R=0.49). Specifically, CD8 cells (R=0.97), endocrine cells (R = 0.93) and fibers (r = 0.89) showed a strong positive correlation (**Figure 3C**). Therefore, the cyan module was selected as the hub module, and the hub gene was extracted from it by using the selection criteria cor.MM>0.7 and cor.GS>0.4.

### 3.3 hub genes of cyan module and Single cell sequencing analysis

**Figure 4:**
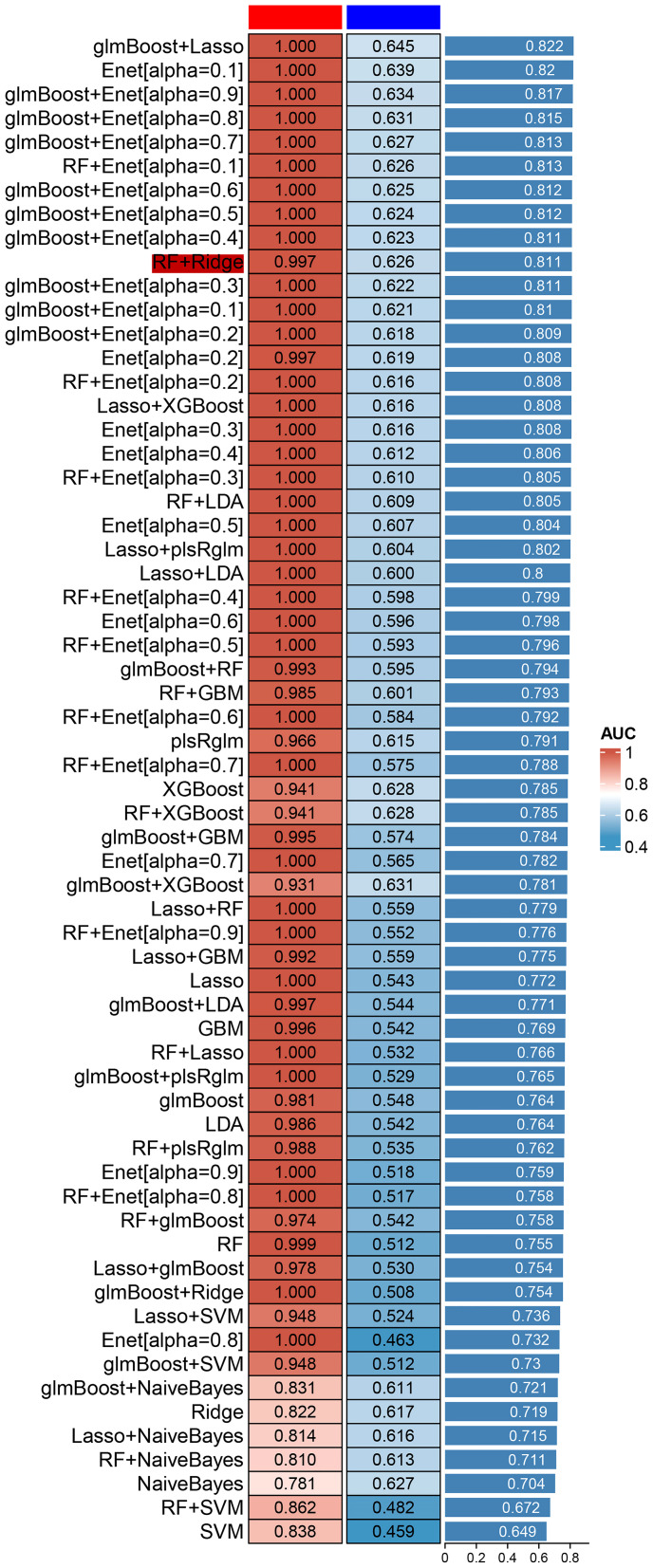
Common machine learning algorithms in pairs and evaluated the diagnostic ability of each feature gene in predicting ASD progression in the internal dataset through calibration curve and subject operating characteristic (ROC) curve analysis.

**Figure 5:**
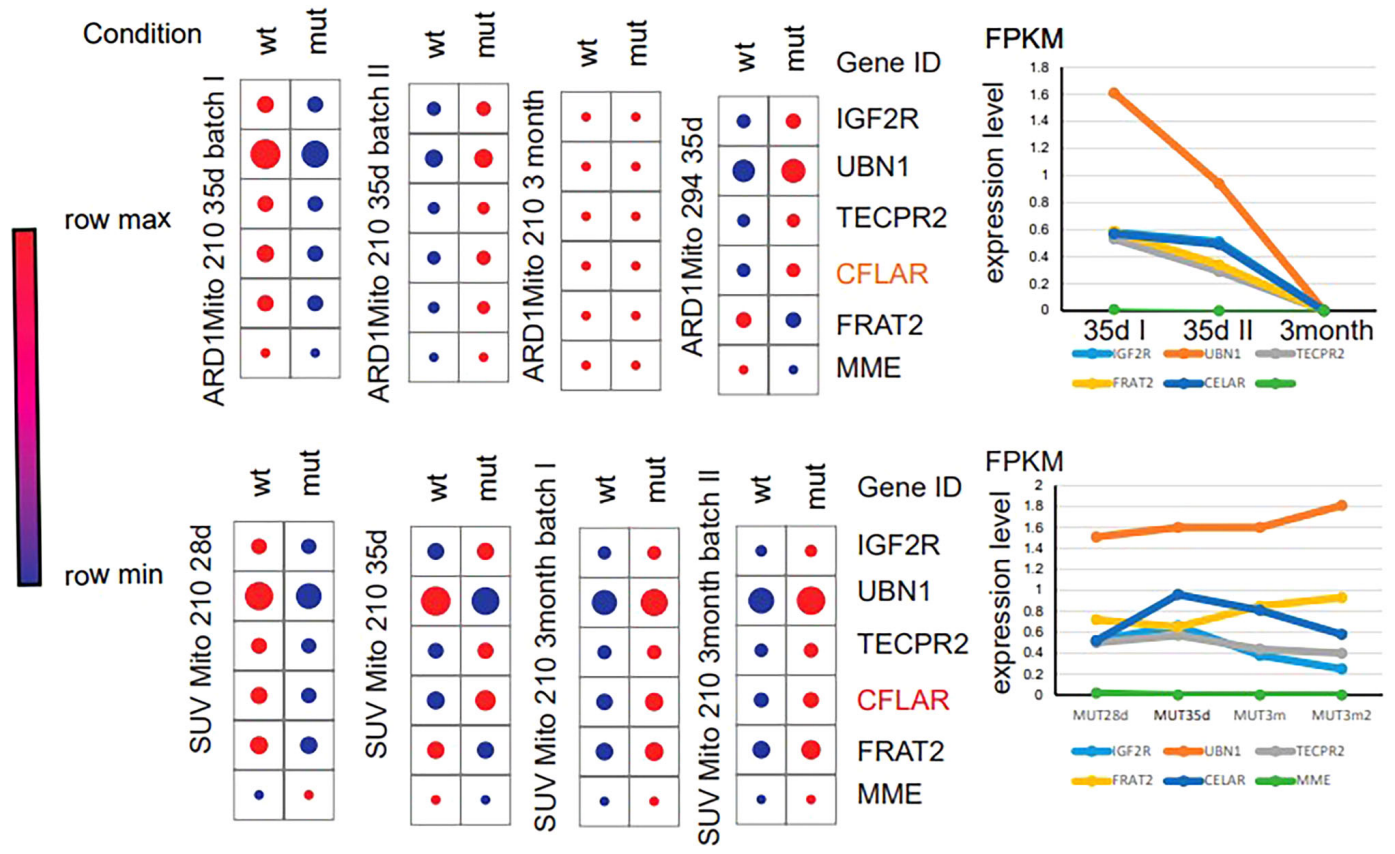
Key gene expression levels at different time nodes.

In order to further clarify the diagnostic and therapeutic effects of feature genes on ASD, we combined common machine learning algorithms in pairs and evaluated the diagnostic ability of each feature gene in predicting ASD progression in the internal dataset through calibration curve and subject operating characteristic (ROC) curve analysis. The AUC values of pairwise combination in the training dataset ranged from 0.649 to 0.822, and we chose the most appropriate RF+Ridge (AUC=0.811) as the final model (**Figure 4**; **Supplementary Table 1**). Subsequently, we compared the expression of key genes in wild-type and mutant ASD, and tracked the changes in key genes according to the different ages of the patients. The single-cell sequencing results indicate that the transcriptome fragments of the 47 core genes exhibit a consistent trend (**Figure 5**).

### 3.4 Estimated genetic correlation

Genome-wide single nucleotide polymorphism (SNP)-based heritability (eri was estimated to for ASD and Neutrophils. Then, we found a significant positive genetic correlation between them ( $\hat{\text{r}}$ g = 0.123, P = 0.0003) by cross-trait genetic correlation analysis. This link implies a possible common genetic cause between them. Hence, it is necessary to further investigate the genetic mechanism.

### 3.5 Shared associated pleiotropic regions

Subsequently, pleiotropy analysis we sued by PLACO and CPASSOC. Therefore, PLACO analysis identified 3187 statistically significant regions (P < 5E-08) (**Supplementary Table S2**), with ASD and Neutrophils. Similarly, we found that 13501 genes were statistically significant (P < 5E-08) (**Supplementary Table S3**), with ASD and Neutrophils. In the end, we found that PLACO and CPASSOC identified a total of 16 potential pleiotropic regions (**Supplementary Table 4**).

### 3.6 Functional analysis for pleiotropic regions

First, we performed gene enrichment analysis using FUMA for the 16 pleiotropic regions identified. And the EQTL results indicated that the differentially expressed genes were predominantly enriched in tissues such as pancreas, liver, heart, blood, brain, and muscle based on the analysis of expression levels (**Supplementary Table 5**).

### 3.7 Expression levels of hub genes

**Figure 6:**
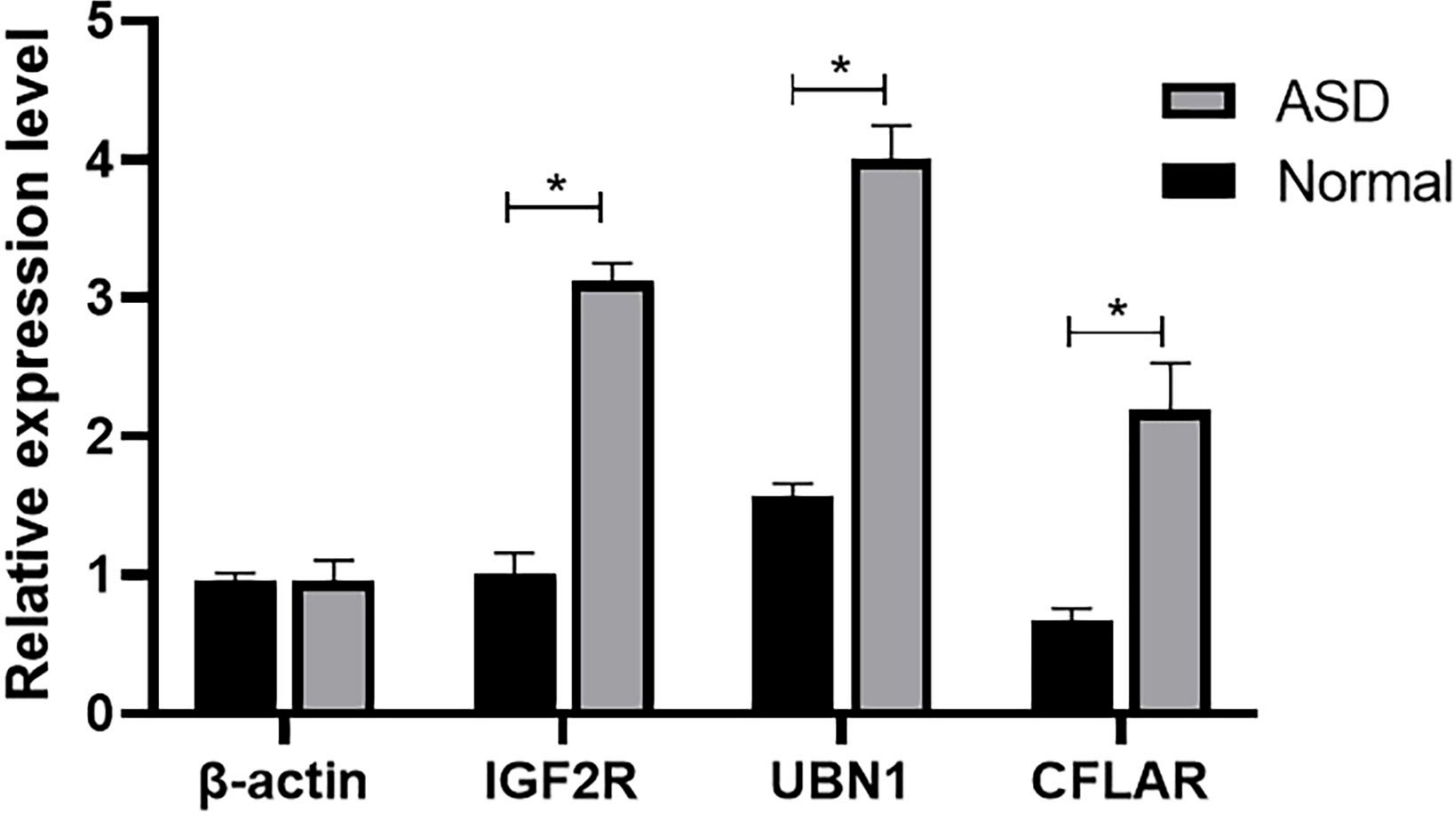
The mRNA level of β-actin, IGF2R, UBN1, and CFLAR was detected by qRT-PCR (*P < 0.05).

The comparison of serum mRNA expression levels between patients with ASD and the normal population shows that, β-actin is basically similar, while the mRNA levels of IGF2R, UBN1 and CFLAR in plasma of children with ASD showed a significant downward trend, and the difference was significant (*P*<0.05), suggesting that the surge of transcriptome had an impact on the development of nervous system (**Figure 6**).

## 4 Discussion

ASD is a neurodevelopmental condition characterized by challenges in social interaction, verbal and nonverbal communication, limited interests and behaviors, and repetitive patterns (26, 27). These difficulties hinder the ability of children with ASD to adapt to social and educational settings, impeding their development and quality of life. Detecting and treating ASD early is challenging due to its elusive early symptomatology, and untreated ASD can result in cognitive impairment and compromise learning, daily functioning, and self-care abilities (28, 29). There is a consensus among experts that further elucidation of diagnostic and treatable biomarkers of ASD is essential. Therefore, accurate characterization and identification of efficient biomarkers are vital for facilitating early diagnosis and successful treatment.

Interestingly, immunoinfiltration analysis showed that inflammatory cells represented by neutrophils were closely related to the occurrence of ASD. Subsequent WGCNA analysis further supported the significant correlation between neutrophils and ASD. It should be noted that we calculated the WGCNA of NK cells, dendritic cells, monocytes and lymphocytes respectively, and the results all indicated that there was no proper classification. Therefore, the WGCNA of neutrophils is the most critical. To further analyze the genomic characteristics of ASD subgroups, we utilized WGCNA to construct a co-expression network for the merged data sets. Through this approach, we discovered that the cyan module was notably consistent, which further substantiates our assumption. A metabolic-related enrichment analysis reveals that the most abundant cyan modules are chiefly concentrated in carbohydrate metabolism, particularly the TCA cycle. These metabolic processes mainly occur in the mitochondrial region. Conversely, any malfunctioning of mitochondria may result in calcium channel imbalance, increased oxidative stress, and reduced ability to cope with reactive oxygen species. Ultimately, this can lead to neuronal damage during early childhood development and can directly influence the social and psychological well-being of ASD patients.

In this paper, we use a new method PLACO to determine the common pleiotropic or common variation between inflammatory level represented by neutrophils and ASD, and show how it can be well applied to related traits or traits in sample overlap research. The results suggest that the high level of neutrophil infiltration combined with six core genes has a direct positive impact on the occurrence of ASD, which greatly increases the probability of fetal ASD. Machine learning algorithms, such as support vector machine, svm), random forest, RF) and proximity algorithm, have been widely used in clinic to make intelligent prediction and decision by automatically learning experience from data, which has played an important role in improving the diagnosis accuracy and prognosis prediction of diseases, and has also been widely reported in the field of nervous system diseases. But the related research is more based on the simple use of a single learning algorithm, rather than the joint application of multiple machine learning algorithms. We combined the above methods and calculated their accuracy, and finally found that RF+Ridge joint prediction has the highest value. In our study, the highest accuracy of machine learning prediction can reach 0.811, which indicates that the model of this study can deeply combine the metabolism-related genes and inflammatory cell subtypes of patients and achieve the purpose of accurate diagnosis. Crucially, we use the above two machines learning algorithms to further decrease the number of central genes. Finally, six central genes were identified, including IGF2R, UBN1, TECPR2, CFLAR, FRAT2, and MME.

**Figure 7:**
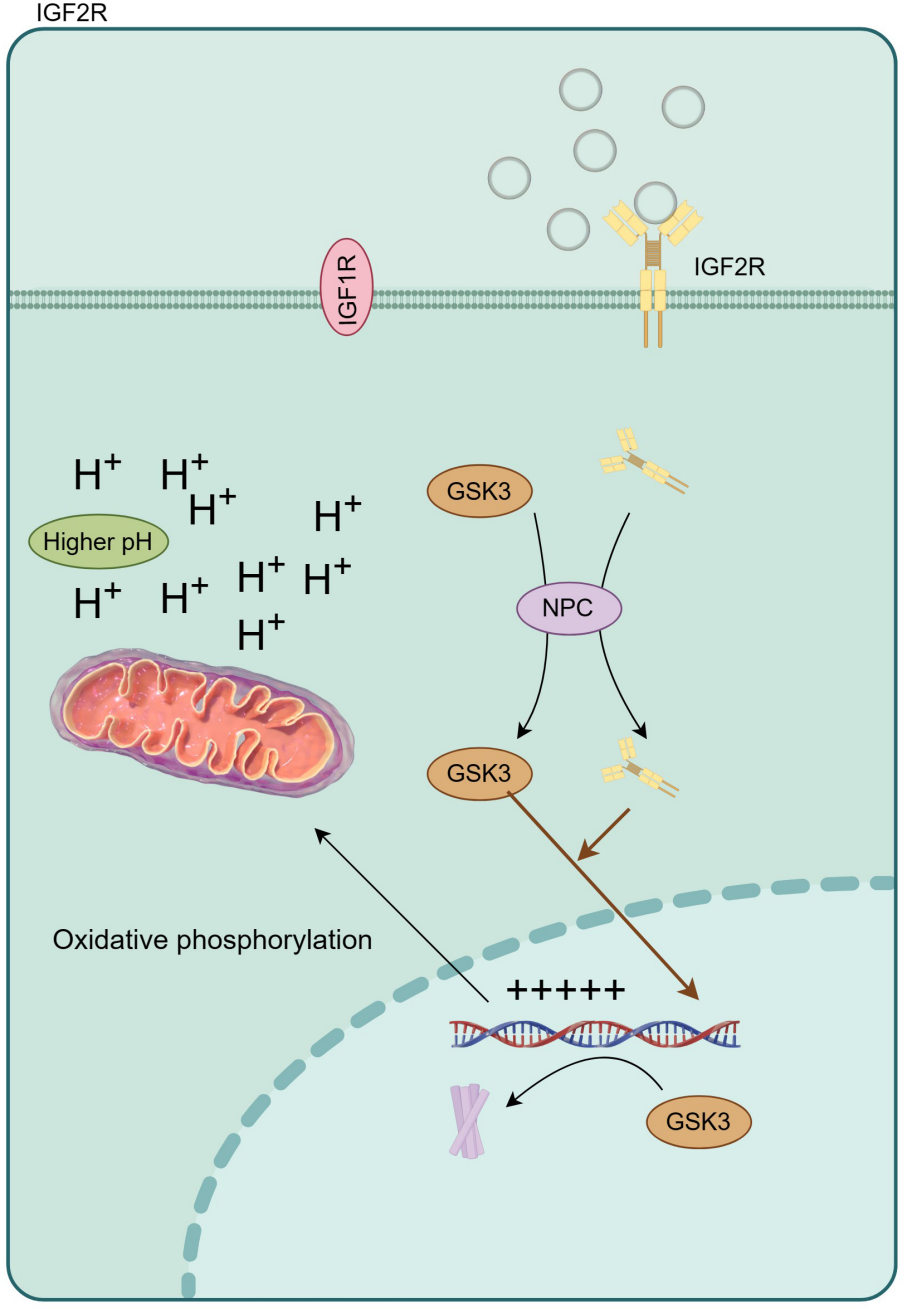
IGF2R regulates oxidative phosphorylation by changing intracellular pH, and then affects the expression of inflammatory factors.

The aberrant activation of signaling cascades mediated by insulin and the insulin-like growth factor (IGF) family has been implicated in the pathophysiology of numerous ailments, including diabetes, cancer, obesity, neurodegenerative disorders, and musculoskeletal conditions (30–33). Within this extensive family, the cation-independent mannose 6-phosphate receptor/insulin-like growth factor II receptor (IGF2R) is commonly referred to as the “scavenging receptor.” Its primary function involves stabilizing local IGF levels through internalization and lysosomal degradation (**Figure 7**). In the mammalian cerebral cortex, IGF2 plays a critical role as an antisense transcript, contributing to neuron-specific epigenetic modifications associated with the lineage of neural stem cells (34). In this study, the most prominent disparity in hub genes manifests in UBN1, acting as a constituent of the HIRA histone chaperone complex, primarily tasked with binding and safeguarding basic histones against non-specific interactions prior to their deposition into nucleosomes. During embryonic development, the hira-1 mutation, particularly in UBN1, incites mitochondrial stress, thereby potentially giving rise to delayed impairments (35, 36). Notably, reported delayed human diseases resulting from UBN1 overexpression encompass Alzheimer’s disease, Parkinson’s disease, and type 2 diabetes. Crucially, especially with regard to intellectual development, UBN1 plays a pivotal role in preserving alkaline histones. Furthermore, our subsequent qPCR findings indicate a significant increase in UBN1’s serum free mRNA levels in ASD children, aligning with the lower levels observed in the mut group through single-cell sequencing. In this study, the most significant is the significant increase of CFLAR, especially when the blood neutrophils of ASD patients are maintained at a high level, suggesting the potential risk of neonatal higher CFLAR inducing ASD in inflammatory response.

As far as we know, this is the first study to classify ASD from the perspective of the methods. The screening and verification of distinctive genes provide potential molecular targets for further investigating the mechanism of ASD. However, there are some shortcomings in this study. First, the comparison of characteristic genes only comes from the blood of human volunteers, and the sample size is small. Secondly, there is a lack of in-depth mechanism exploration based on experimental animals. Finally, including death records in the included population may lead to some bias in our results, and the grid analysis of hub genes needs to be further validated *in vitro* and *in vivo* experiments. Therefore, the above issues are also a hallmark of our future research.

In conclusion, our study yields unprecedented insights into the molecular and genetic heterogeneity of ASD through a comprehensive bioinformatics analysis. Furthermore, six essential genes, namely IGF2R, UBN1, TECPR2, CFLAR, FRAT2, and MME, have been successfully identified and validated. These valuable findings hold significant implications for tailoring personalized ASD therapies. Ultimately, our endeavor aims to enhance our comprehension of the fundamental mechanisms governing ASD and facilitate the development of efficacious treatments for this intricate disorder.

## Data availability statement

The original contributions presented in the study are included in the article/**Supplementary Material**. Further inquiries can be directed to the corresponding authors.

## Ethics statement

The studies involving humans were approved by Biomedical Basic Research Ethics Committee of Xiangyang No.1 People’s Hospital (XYYYE20240052). The studies were conducted in accordance with the local legislation and institutional requirements. Written informed consent for participation in this study was provided by the participants’ legal guardians/next of kin.

## Author contributions

JM: Conceptualization, Writing – review & editing. DL: Data curation, Formal analysis, Software, Writing – original draft. JZ: Software, Writing – original draft. XF: Data curation, Methodology, Writing – review & editing. DB: Conceptualization, Writing – review & editing.

## References

[1] Frasch MG Yoon BJ Helbing DL Snir G Antonelli MC Bauer R. Autism spectrum disorder: A neuro-immunometabolic hypothesis of the developmental origins. Biology. (2023) 12(7):914. doi: 10.3390/biology12070914 37508346 PMC10375982

[2] Suzuki K Sugihara G Ouchi Y Nakamura K Futatsubashi M Takebayashi K. Microglial activation in young adults with autism spectrum disorder. JAMA Psychiatry. (2013) 70:49–58. doi: 10.1001/jamapsychiatry.2013.272 23404112

[3] Wang Y Yu S Li M. Neurovascular crosstalk and cerebrovascular alterations: an underestimated therapeutic target in autism spectrum disorders. Front Cell Neurosci. (2023) 17:1226580. doi: 10.3389/fncel.2023.1226580 37692552 PMC10491023

[4] Shan J Gu Y Zhang J Hu X Wu H Yuan T. A scoping review of physiological biomarkers in autism. Front Neurosci. (2023) 17:1269880. doi: 10.3389/fnins.2023.1269880 37746140 PMC10512710

[5] Zhang J Ji G Gao X Guan J. Single-nucleus gene and gene set expression-based similarity network fusion identifies autism molecular subtypes. BMC Bioinf. (2023) 24:142. doi: 10.1186/s12859-023-05278-0 PMC10091652 37041460

[6] Lian P Cai X Wang C Liu K Yang X Wu Y. Identification of metabolism-related subtypes and feature genes in Alzheimer's disease. J Trans Med. (2023) 21:628. doi: 10.1186/s12967-023-04324-y PMC10504766 37715200

[7] Fatima M Srivastav S Mondal AC. Prenatal stress and depression associated neuronal development in neonates. Int J Dev Neurosci Off J Int Soc Dev Neurosci. (2017) 60:1–7. doi: 10.1016/j.ijdevneu.2017.04.001 28389369

[8] Naseh M Vatanparast J Rafati A Bayat M Haghani M. The emerging role of FTY720 as a sphingosine 1-phosphate analog for the treatment of ischemic stroke: The cellular and molecular mechanisms. Brain Behav. (2021) 11:e02179. doi: 10.1002/brb3.2179 33969931 PMC8213944

[9] Keshavarz S Nemati M Saied Salehi M Naseh M. The impact of anesthetic drugs on hemodynamic parameters and neurological outcomes following temporal middle cerebral artery occlusion in rats. Neuroreport. (2023) 34:199–204. doi: 10.1097/wnr.0000000000001863 36789841 PMC10516172

[10] Chen C Zong S Wang Z Yang R Guo Y Wang Y. FTY720 attenuates LPS-induced inflammatory bone loss by inhibiting osteoclastogenesis via the NF-κB and HDAC4/ATF pathways. J Immunol Res. (2023) 2023:8571649. doi: 10.1155/2023/8571649 36644540 PMC9839404

[11] Racle J Gfeller D. EPIC: A tool to estimate the proportions of different cell types from bulk gene expression data. Methods Mol Biol (Clifton NJ). (2020) 2120:233–48. doi: 10.1007/978-1-0716-0327-7_17 32124324

[12] Subramanian A Tamayo P Mootha VK Mukherjee S Ebert BL Gillette MA. Gene set enrichment analysis: a knowledge-based approach for interpreting genome-wide expression profiles. Proc Natl Acad Sci United States America. (2005) 102:15545–50. doi: 10.1073/pnas.0506580102 PMC1239896 16199517

[13] Finotello F Mayer C Plattner C Laschober G Rieder D Hackl H. Molecular and pharmacological modulators of the tumor immune contexture revealed by deconvolution of RNA-seq data. Genome Med. (2019) 11:34. doi: 10.1186/s13073-019-0638-6 31126321 PMC6534875

[14] Li B Liu JS Liu XS. Revisit linear regression-based deconvolution methods for tumor gene expression data. Genome Biol. (2017) 18:127. doi: 10.1186/s13059-017-1256-5 28679386 PMC5499010

[15] Newman AM Liu CL Green MR Gentles AJ Feng W Xu Y. Robust enumeration of cell subsets from tissue expression profiles. Nat Methods. (2015) 12:453–7. doi: 10.1038/nmeth.3337 PMC4739640 25822800

[16] Zhao Z Luo Q Liu Y. Multi-level integrative analysis of the roles of lncRNAs and differential mRNAs in the progression of chronic pancreatitis to pancreatic ductal adenocarcinoma. BMC Genomics. (2023) 24(1):101. doi: 10.1186/s12864-023-09209-4 36879212 PMC9990329

[17] Tang C Deng L Luo Q He G. Identification of oxidative stress-related genes and potential mechanisms in atherosclerosis. Front Genet. (2023) 13:998954. doi: 10.3389/fgene.2022.998954 36685865 PMC9845256

[18] Lu Q Li B Ou D Erlendsdottir M Powles RL Jiang T. A powerful approach to estimating annotation-stratified genetic covariance via GWAS summary statistics. Am J Hum Genet. (2017) 101:939–64. doi: 10.1016/j.ajhg.2017.11.001 PMC5812911 29220677

[19] Bulik-Sullivan BK Loh PR Finucane HK Ripke S Yang J Patterson N. LD Score regression distinguishes confounding from polygenicity in genome-wide association studies. Nat Genet. (2015) 47:291–5. doi: 10.1038/ng.3211 PMC4495769 25642630

[20] Yuan J Zhang J Luo Q Peng L. Effects of nonalcoholic fatty liver disease on sarcopenia: evidence from genetic methods. Sci Rep. (2024) 14:2709. doi: 10.1038/s41598-024-53112-1 38302636 PMC10834579

[21] Ray D Chatterjee N. A powerful method for pleiotropic analysis under composite null hypothesis identifies novel shared loci between Type 2 Diabetes and Prostate Cancer. PloS Genet. (2020) 16:e1009218. doi: 10.1371/journal.pgen.1009218 33290408 PMC7748289

[22] Huang YT. Variance component tests of multivariate mediation effects under composite null hypotheses. Biometrics. (2019) 75:1191–204. doi: 10.1111/biom.13073 31009061

[23] Zhu X Feng T Tayo BO Liang J Young JH Franceschini N. Meta-analysis of correlated traits via summary statistics from GWASs with an application in hypertension. Am J Hum Genet. (2015) 96:21–36. doi: 10.1016/j.ajhg.2014.11.011 25500260 PMC4289691

[24] Zhu Z Anttila V Smoller JW Lee PH. Statistical power and utility of meta-analysis methods for cross-phenotype genome-wide association studies. PloS One. (2018) 13:e0193256. doi: 10.1371/journal.pone.0193256 29494641 PMC5832233

[25] Watanabe K Taskesen E van Bochoven A Posthuma D. Functional mapping and annotation of genetic associations with FUMA. Nat Commun. (2017) 8:1826. doi: 10.1038/s41467-017-01261-5 29184056 PMC5705698

[26] Li N Yang J Zhang J Liang C Wang Y Chen B. Correlation of gut microbiome between ASD children and mothers and potential biomarkers for risk assessment. Genomics Proteomics Bioinf. (2019) 17:26–38. doi: 10.1016/j.gpb.2019.01.002 PMC6520911 31026579

[27] Crowell JA Keluskar J Gorecki A. Parenting behavior and the development of children with autism spectrum disorder. Compr Psychiatry. (2019) 90:21–9. doi: 10.1016/j.comppsych.2018.11.007 30658339

[28] Olson L Kinnear M Chen B Reynolds S Ibarra C Wang T. Socioeconomic factors account for variability in language skills in preschoolers with autism spectrum disorders. J Dev Behav Pediatr JDBP. (2021) 42:101–8. doi: 10.1097/dbp.0000000000000870 PMC7864097 33027104

[29] Bal VH Fok M Lord C Smith IM Mirenda P Szatmari P. Predictors of longer-term development of expressive language in two independent longitudinal cohorts of language-delayed preschoolers with Autism Spectrum Disorder. J Child Psychol psychiatry Allied disciplines. (2020) 61:826–35. doi: 10.1111/jcpp.13117 PMC7028445 31429087

[30] Feng CC Pandey S Lin CY Shen CY Chang RL Chang TT. Cardiac apoptosis induced under high glucose condition involves activation of IGF2R signaling in H9c2 cardiomyoblasts and streptozotocin-induced diabetic rat hearts. Biomedicine pharmacotherapy = Biomedecine pharmacotherapie. (2018) 97:880–5. doi: 10.1016/j.biopha.2017.11.020 29136764

[31] Torrente Y Bella P Tripodi L Villa C Farini A. Role of insulin-like growth factor receptor 2 across muscle homeostasis: implications for treating muscular dystrophy. Cells. (2020) 9(2):441. doi: 10.3390/cells9020441 32075092 PMC7072799

[32] Chen RJ Wu HC Chang MH Lai CH Tien YC Hwang JM. Leu27IGF2 plays an opposite role to IGF1 to induce H9c2 cardiomyoblast cell apoptosis via Galphaq signaling. J Mol Endocrinol. (2009) 43:221–30. doi: 10.1677/jme-08-0121 19556392

[33] Chu CH Tzang BS Chen LM Kuo CH Cheng YC Chen LY. IGF-II/mannose-6-phosphate receptor signaling induced cell hypertrophy and atrial natriuretic peptide/BNP expression via Galphaq interaction and protein kinase C-alpha/CaMKII activation in H9c2 cardiomyoblast cells. J Endocrinol. (2008) 197:381–90. doi: 10.1677/joe-07-0619 18434368

[34] Alberini CM. IGF2 in memory, neurodevelopmental disorders, and neurodegenerative diseases. Trends Neurosci. (2023) 46:488–502. doi: 10.1016/j.tins.2023.03.007 37031050 PMC10192130

[35] Ricketts MD Dasgupta N Fan J Han J Gerace M Tang Y. The HIRA histone chaperone complex subunit UBN1 harbors H3/H4- and DNA-binding activity. J Biol Chem. (2019) 294:9239–59. doi: 10.1074/jbc.RA119.007480 PMC6556585 31040182

[36] Burgess RJ Zhang Z. Histone chaperones in nucleosome assembly and human disease. Nat Struct Mol Biol. (2013) 20:14–22. doi: 10.1038/nsmb.2461 23288364 PMC4004355

